# Poly[tetrakis(μ-1,1,1,3,3,3-hexafluoropropan-2-olato)iron(II)dipotassium]

**DOI:** 10.1107/S1600536813034946

**Published:** 2014-01-08

**Authors:** Andrew P. Purdy, Ray J. Butcher

**Affiliations:** a Naval Research Laboratory, Chemistry Division, Code 6100, 4555 Overlook Av, SW, Washington DC 20375, USA; b Department of Chemistry, Howard University, 525 College Street NW, Washington DC, 20059, USA

## Abstract

The title compound, [K_2_Fe{OCH(CF_3_)_2_}_4_]_*n*_, was formed from the reaction of potassium hexa­fluoro­isopropoxide with iron(II) chloride in toluene. The Fe^II^ atom has a highly distorted tetra­hedral coordination environment. All four of the non-equivalent hexa­fluoro­isoprop­oxy O atoms link the Fe^II^ atoms to one of the K^+^ atoms in an alternating chain of Fe—O—K—O fused four-membered rings, with K—Fe distances of 3.715 (2) and 3.717 (2) Å. This K^+^ atom is also bridged to eight of the F atoms. The other K^+^ atom is bonded to only two of the O atoms, but has seven short K⋯F contacts, one of which links the chains into a three-dimensional arrangement. Weak hydrogen bonding between the lone H atoms on the hexa­fluoro­isoprop­oxy groups and F atoms is also present. The crystal studied was refined as an inversion twin.

## Related literature   

For alkali or alkaline earth metal fluoro­alkoxides with short F—*A* distances, see: Zheng *et al.* (2009 ▶); Yamashita *et al.* (2005 ▶); Bernhardt *et al.* (2007 ▶); Purdy & George (1991 ▶, 1994 ▶); Samuels *et al.* (1993 ▶); Purdy *et al.* (1991 ▶). For iron(II) fluoro­alkoxides, see: Konefal *et al.* (1986 ▶); Cantalupo *et al.* (2010 ▶, 2012 ▶). 
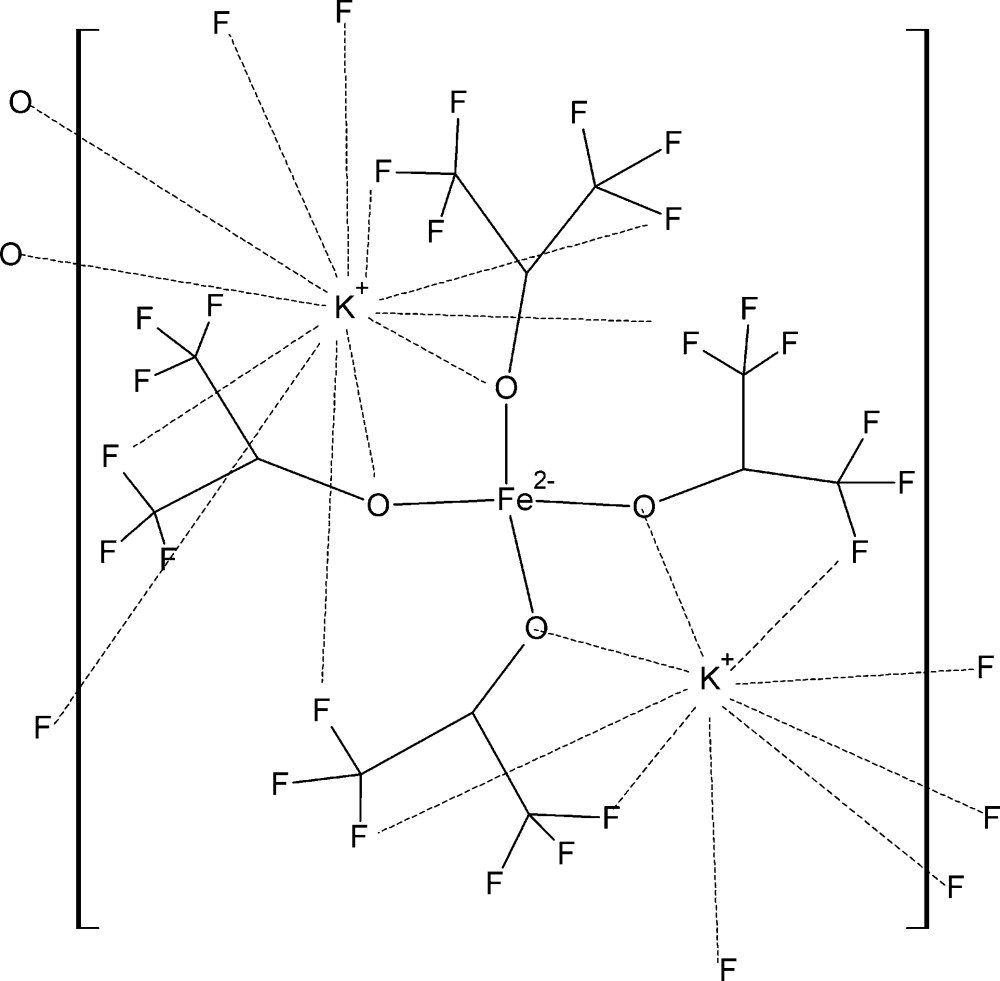


## Experimental   

### 

#### Crystal data  

[FeK_2_(C_3_HF_6_O)_4_]
*M* *_r_* = 802.20
Orthorhombic, 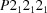

*a* = 9.7368 (2) Å
*b* = 13.4345 (4) Å
*c* = 18.3933 (4) Å
*V* = 2406.02 (10) Å^3^
*Z* = 4
Cu *K*α radiation
μ = 10.15 mm^−1^
*T* = 123 K
0.43 × 0.21 × 0.17 mm

#### Data collection  

Agilent Xcalibur (Ruby, Gemini) diffractometer
Absorption correction: multi-scan (*CrysAlis PRO*; Agilent, 2012 ▶) *T*_min_ = 0.254, *T*_max_ = 1.000
16099 measured reflections
4923 independent reflections
4190 reflections with *I* > 2σ(*I*)
*R*_int_ = 0.100

#### Refinement  

*R*[*F* ^2^ > 2σ(*F* ^2^)] = 0.068
*wR*(*F* ^2^) = 0.182
*S* = 1.02
4923 reflections
389 parameters
144 restraints
H-atom parameters constrained
Δρ_max_ = 1.63 e Å^−3^
Δρ_min_ = −1.29 e Å^−3^
Absolute structure: Refined as an inversion twin
Absolute structure parameter: 0.205 (11)

### 

Data collection: *CrysAlis PRO* (Agilent, 2012 ▶); cell refinement: *CrysAlis PRO*; data reduction: *CrysAlis PRO*; program(s) used to solve structure: *SHELXS97* (Sheldrick, 2008 ▶); program(s) used to refine structure: *SHELXL2013* (Sheldrick, 2008 ▶); molecular graphics: *SHELXTL* (Sheldrick, 2008 ▶); software used to prepare material for publication: *SHELXTL*.

## Supplementary Material

Crystal structure: contains datablock(s) I, New_Global_Publ_Block. DOI: 10.1107/S1600536813034946/nk2215sup1.cif

Structure factors: contains datablock(s) I. DOI: 10.1107/S1600536813034946/nk2215Isup2.hkl

CCDC reference:

Additional supporting information:  crystallographic information; 3D view; checkCIF report

## Figures and Tables

**Table 1:** Hydrogen-bond geometry (Å, °)

*D*—H⋯*A*	*D*—H	H⋯*A*	*D*⋯*A*	*D*—H⋯*A*
C1*A*—H1*AA*⋯F3*B* ^i^	1.00	2.54	3.236 (10)	126
C1*B*—H1*BA*⋯F2*C*	1.00	2.56	3.199 (11)	122
C1*C*—H1*CA*⋯F4*D* ^ii^	1.00	2.56	3.201 (11)	122
C1*D*—H1*DA*⋯F2*A* ^iii^	1.00	2.46	3.361 (11)	150
C1*D*—H1*DA*⋯F3*A*	1.00	2.42	3.093 (11)	124

Symmetry codes: (i) 
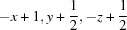
; (ii) 
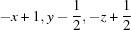
; (iii) 
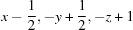
.

**Fig. 1.**
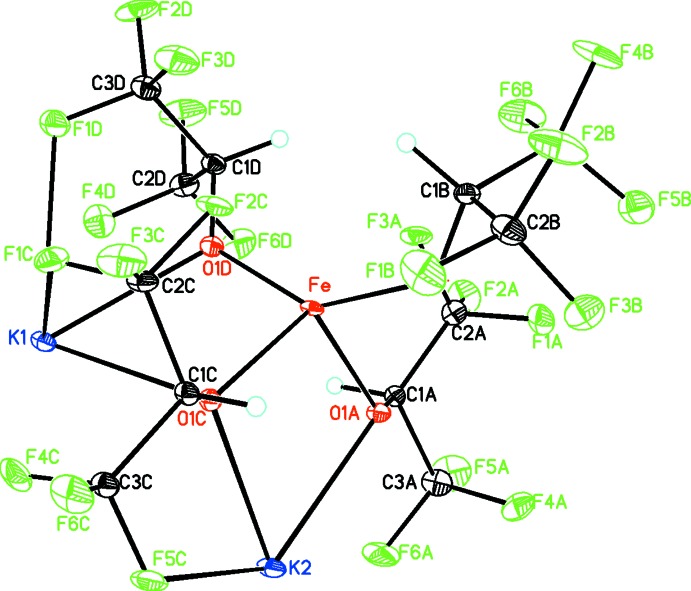
Structure of the repeat unit of [K2Fe(OCH(CF3)2)4]. Ellipsoids are at the 30% probability level.

**Fig. 2.**
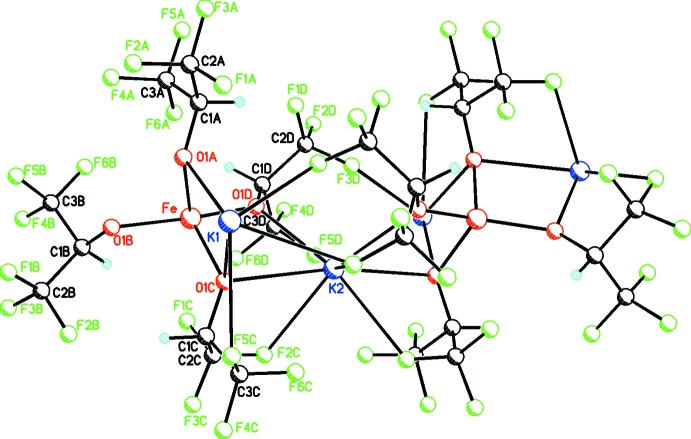
Diagram showing the potassium coordination environment.

**Fig. 3.**
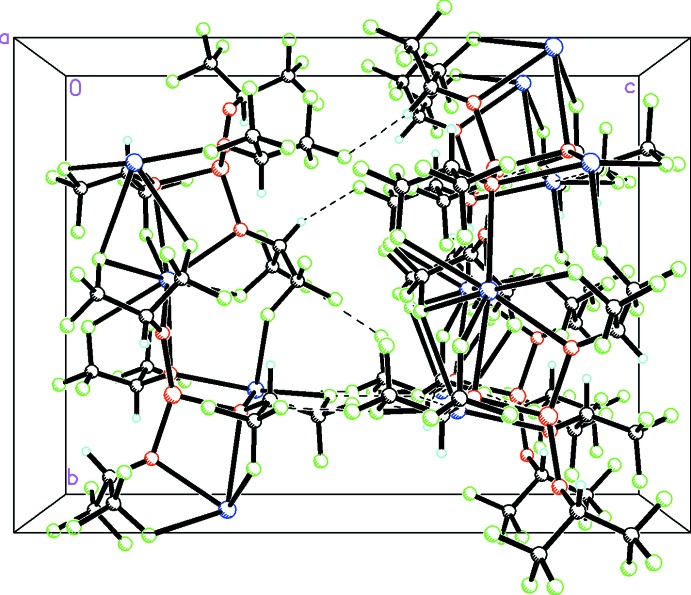
Packing diagram depicting some of the hydrogen bonding.

## supplementary crystallographic information

### 1. Comment

The iron atoms have a highly distorted tetrahedral coordination by the four O atoms of the hexafluoroisopropoxide ligands, with the O—Fe—O angles ranging from 93.9 (2) to 143.1 (2) °. The two longest Fe—O bonds (2.047 (6) and 2.048 (6) Å) are to the O atoms O1A and O1C, which are also bridged to both K1 and K2. The shorter Fe—O bonds (1.948 (6) and 1.950 (6) Å) are to O1B and O1D, which are bridged only to K1 atoms. While the metal atoms are linked in a zigzag chain along b by coordination to these O atoms, coordination of the fluorine atoms to the potassium atoms completes the potassium coordination spheres. K2 is 9-coordinate and is linked to O1A and O1C (2.689 (6) and 2.698 (7) Å), and 7 fluorine atoms on the HFIP groups, with K—F contacts ranging from 2.798 (8) to 3.088 (7) Å. One fluorine atom, F6C, links K2 to a symmetry equivalent chain. K1 is 12-coordinate with two short K—O bonds of 2.674 (6) and 2.692 (7) Å and two longer K—O bonds of 2.891 (6) and 2.863 (7). The eight K—F contacts range from 2.861 (6) to 3.221 (7) Å. Close contacts between alkali or alkaline earth cations and the fluorine atoms of a fluoroalkoxy ligand are very common and occur in many compounds that have been structurally characterized. Some examples include Na_2_Cu(OCH(CF_3_)_2_)_4_ (Purdy, *et al.* 1991), BaCu(OCH(CF_3_)_2_)_4_(THF)_4_ (Purdy and George, 1994), (18-Crown-6)-(1,1-bis(trifluoromethyl)-1*H*-anthra(1,9-*bc*)furan-10-olato-F, O)-potassium (Yamashita, *et al.*, 2005), and some potassium perfluoroalkoxyborates (Bernhardt, *et al.*, 2007). The nature of the bonding between fluorines on fluoroalkoxy groups and electropositive metal ions was explained by a combination of orbital overlap and electrostatics (Samuels *et al.*, 1993). Weak C—H···F hydrogen bonding is also present with all the hexafluoroisopropoxy protons.

### 2. Experimental

All manipulations were performed under inert atmosphere (Ar). Sublimed FeCl_2_ (0.66 g, 5.21 mmol) and potassium hexafluoroisopropoxide (2.16 g, 10.48 mmol) were combined in 20 ml toluene in an H-tube equipped with a fine frit, and sonicated for a week. Mixture was filtered and rewashed 3x with recondensed hot toluene. Some product is slightly soluble in toluene and that solution was allowed to evaporate slowly in a flask in the drybox. Pale yellow crystals resulted, and a crystal of the title compound was obtained from this sample. (The crystals looked pale yellow in bulk but individual crystals appeared almost colorless.) A total of 0.56 g was isolated. NMR: ^19^F (THF solution, CFCl_3_ ref) -37.89.

### 3. Refinement

Crystal data, data collection and structure refinement details are summarized in Table 1.

### Crystal data

**Table:**

[FeK_2_(C_3_HF_6_O)_4_]	*D*_x_ = 2.215 Mg m^−^^3^
*M**_r_* = 802.20	Cu *K*α radiation, λ = 1.54184 Å
Orthorhombic, *P*2_1_2_1_2_1_	Cell parameters from 8115 reflections
*a* = 9.7368 (2) Å	θ = 3.3–75.5°
*b* = 13.4345 (4) Å	µ = 10.15 mm^−^^1^
*c* = 18.3933 (4) Å	*T* = 123 K
*V* = 2406.02 (10) Å^3^	Prism, colorless
*Z* = 4	0.43 × 0.21 × 0.17 mm
*F*(000) = 1552	

### Data collection

**Table:**

Agilent Xcalibur (Ruby, Gemini) diffractometer	4923 independent reflections
Radiation source: Enhance (Cu) X-ray Source	4190 reflections with *I* > 2σ(*I*)
Detector resolution: 10.5081 pixels mm^-1^	*R*_int_ = 0.100
ω scans	θ_max_ = 75.7°, θ_min_ = 4.1°
Absorption correction: multi-scan (*CrysAlis PRO*; Agilent, 2012)	*h* = −12→8
*T*_min_ = 0.254, *T*_max_ = 1.000	*k* = −16→16
16099 measured reflections	*l* = −22→17

### Refinement

**Table:**

Refinement on *F*^2^	Hydrogen site location: inferred from neighbouring sites
Least-squares matrix: full	H-atom parameters constrained
*R*[*F*^2^ > 2σ(*F*^2^)] = 0.068	*w* = 1/[σ^2^(*F*_o_^2^) + (0.1306*P*)^2^] where *P* = (*F*_o_^2^ + 2*F*_c_^2^)/3
*wR*(*F*^2^) = 0.182	(Δ/σ)_max_ < 0.001
*S* = 1.02	Δρ_max_ = 1.63 e Å^−^^3^
4923 reflections	Δρ_min_ = −1.29 e Å^−^^3^
389 parameters	Absolute structure: Refined as an inversion twin
144 restraints	Absolute structure parameter: 0.205 (11)

### Special details

**Table:**

Experimental. Empirical absorption correction using spherical harmonics, implemented in SCALE3 ABSPACK scaling algorithm.
Geometry. All e.s.d.'s (except the e.s.d. in the dihedral angle between two l.s. planes) are estimated using the full covariance matrix. The cell e.s.d.'s are taken into account individually in the estimation of e.s.d.'s in distances, angles and torsion angles; correlations between e.s.d.'s in cell parameters are only used when they are defined by crystal symmetry. An approximate (isotropic) treatment of cell e.s.d.'s is used for estimating e.s.d.'s involving l.s. planes.
Refinement. Refined as a 2-component inversion twin. The structure was a racemic twin with a BASF component of 0.20906. In spite of the fact that the data was collected at -150 ° C the CF_3_ groups had some problems. Disorder was looked at without success but the best solution was to restrain using ISOR compound for some (but not all) *F*'s.

### Fractional atomic coordinates and isotropic or equivalent isotropic displacement parameters (Å^2^)

**Table:**

	*x*	*y*	*z*	*U*_iso_*/*U*_eq_	
Fe	0.41997 (13)	0.23850 (10)	0.28502 (6)	0.0109 (3)	
K1	0.41988 (19)	0.48834 (14)	0.19850 (9)	0.0162 (4)	
K2	0.69671 (19)	0.23877 (16)	0.16065 (9)	0.0196 (4)	
O1A	0.6217 (6)	0.1993 (5)	0.2983 (3)	0.0152 (12)	
C1A	0.6962 (9)	0.2291 (6)	0.3584 (4)	0.0145 (16)	
H1AA	0.6995	0.3035	0.3588	0.017*	
C2A	0.6316 (10)	0.1946 (7)	0.4284 (5)	0.0186 (18)	
C3A	0.8423 (10)	0.1903 (8)	0.3490 (5)	0.024 (2)	
F1A	0.6251 (7)	0.0950 (4)	0.4340 (3)	0.0284 (14)	
F2A	0.6983 (7)	0.2266 (5)	0.4876 (3)	0.0347 (15)	
F3A	0.5028 (6)	0.2283 (5)	0.4341 (3)	0.0272 (13)	
F4A	0.8480 (7)	0.0916 (5)	0.3445 (4)	0.0320 (14)	
F5A	0.9267 (7)	0.2178 (6)	0.4034 (4)	0.0449 (18)	
F6A	0.8971 (6)	0.2258 (6)	0.2876 (4)	0.0378 (16)	
O1B	0.3535 (7)	0.1029 (5)	0.2980 (4)	0.0181 (13)	
C1B	0.2276 (9)	0.0657 (7)	0.3165 (5)	0.0170 (18)	
H1BA	0.1580	0.1204	0.3163	0.020*	
C2B	0.1877 (11)	−0.0141 (8)	0.2603 (5)	0.025 (2)	
C3B	0.2342 (11)	0.0183 (8)	0.3922 (6)	0.026 (2)	
F1B	0.1707 (8)	0.0271 (6)	0.1949 (3)	0.0406 (17)	
F2B	0.0739 (8)	−0.0631 (6)	0.2766 (4)	0.047 (2)	
F3B	0.2873 (7)	−0.0833 (5)	0.2517 (3)	0.0323 (14)	
F4B	0.1165 (8)	−0.0222 (6)	0.4141 (4)	0.0458 (19)	
F5B	0.3307 (8)	−0.0548 (5)	0.3957 (3)	0.0351 (15)	
F6B	0.2697 (9)	0.0858 (6)	0.4414 (4)	0.0445 (19)	
O1C	0.4260 (6)	0.2752 (4)	0.1770 (3)	0.0139 (11)	
C1C	0.3268 (9)	0.2457 (7)	0.1284 (4)	0.0144 (16)	
H1CA	0.3238	0.1713	0.1277	0.017*	
C2C	0.1858 (9)	0.2841 (7)	0.1495 (5)	0.0175 (17)	
C3C	0.3700 (10)	0.2821 (7)	0.0534 (5)	0.0177 (18)	
F1C	0.1787 (6)	0.3837 (5)	0.1504 (3)	0.0304 (14)	
F2C	0.1552 (6)	0.2545 (5)	0.2178 (3)	0.0260 (12)	
F3C	0.0838 (6)	0.2516 (6)	0.1074 (3)	0.0341 (14)	
F4C	0.3812 (7)	0.3802 (5)	0.0496 (3)	0.0325 (15)	
F5C	0.4927 (6)	0.2443 (5)	0.0362 (3)	0.0295 (13)	
F6C	0.2830 (7)	0.2540 (6)	0.0002 (3)	0.0342 (14)	
O1D	0.3927 (7)	0.3754 (5)	0.3173 (3)	0.0165 (12)	
C1D	0.3404 (10)	0.4119 (7)	0.3802 (5)	0.0171 (18)	
H1DA	0.3309	0.3563	0.4160	0.020*	
C2D	0.4377 (10)	0.4903 (8)	0.4112 (5)	0.023 (2)	
C3D	0.1970 (12)	0.4569 (8)	0.3651 (5)	0.024 (2)	
F1D	0.2019 (6)	0.5245 (5)	0.3110 (3)	0.0318 (14)	
F2D	0.1412 (7)	0.5024 (6)	0.4213 (3)	0.0421 (19)	
F3D	0.1113 (7)	0.3866 (6)	0.3436 (4)	0.0391 (17)	
F4D	0.4577 (7)	0.5661 (5)	0.3630 (4)	0.0351 (15)	
F5D	0.3921 (8)	0.5317 (6)	0.4723 (4)	0.046 (2)	
F6D	0.5606 (7)	0.4520 (6)	0.4243 (4)	0.0359 (16)	

### Atomic displacement parameters (Å^2^)

**Table:**

	*U*^11^	*U*^22^	*U*^33^	*U*^12^	*U*^13^	*U*^23^
Fe	0.0062 (5)	0.0223 (7)	0.0041 (5)	0.0004 (5)	−0.0009 (4)	0.0011 (5)
K1	0.0121 (8)	0.0246 (9)	0.0119 (7)	−0.0017 (7)	0.0010 (7)	0.0019 (6)
K2	0.0133 (8)	0.0323 (10)	0.0134 (7)	0.0013 (8)	0.0029 (7)	0.0011 (7)
O1A	0.010 (2)	0.024 (3)	0.011 (2)	0.003 (2)	−0.002 (2)	0.002 (2)
C1A	0.014 (2)	0.0158 (19)	0.0142 (19)	−0.0003 (13)	−0.0017 (13)	0.0004 (13)
C2A	0.018 (3)	0.023 (3)	0.015 (3)	0.000 (3)	−0.006 (3)	−0.002 (3)
C3A	0.015 (4)	0.040 (5)	0.017 (4)	−0.001 (4)	−0.009 (4)	0.000 (4)
F1A	0.041 (4)	0.031 (3)	0.014 (2)	0.001 (3)	−0.003 (2)	0.003 (2)
F2A	0.041 (3)	0.050 (4)	0.012 (2)	0.001 (3)	−0.016 (2)	−0.002 (2)
F3A	0.022 (3)	0.048 (4)	0.012 (2)	0.010 (3)	0.005 (2)	0.002 (2)
F4A	0.019 (3)	0.039 (4)	0.038 (3)	0.009 (3)	−0.004 (3)	−0.002 (3)
F5A	0.021 (3)	0.069 (4)	0.045 (4)	−0.002 (3)	−0.023 (3)	−0.010 (3)
F6A	0.012 (3)	0.062 (4)	0.039 (4)	−0.001 (3)	0.009 (2)	0.008 (3)
O1B	0.010 (2)	0.021 (3)	0.023 (3)	−0.001 (2)	0.004 (2)	0.000 (2)
C1B	0.013 (3)	0.021 (3)	0.017 (3)	0.002 (3)	0.004 (3)	0.001 (3)
C2B	0.020 (5)	0.033 (5)	0.021 (4)	−0.006 (4)	0.000 (4)	0.003 (4)
C3B	0.023 (4)	0.032 (4)	0.022 (3)	−0.001 (3)	0.008 (3)	−0.004 (3)
F1B	0.042 (4)	0.060 (4)	0.019 (3)	−0.006 (3)	−0.013 (3)	0.005 (3)
F2B	0.026 (4)	0.073 (5)	0.042 (4)	−0.031 (4)	0.008 (3)	−0.011 (3)
F3B	0.035 (4)	0.036 (3)	0.026 (3)	0.003 (3)	−0.002 (3)	−0.009 (3)
F4B	0.030 (4)	0.075 (5)	0.033 (3)	−0.017 (3)	0.017 (3)	0.008 (3)
F5B	0.036 (4)	0.046 (4)	0.022 (3)	0.008 (3)	0.000 (3)	0.007 (3)
F6B	0.049 (5)	0.063 (5)	0.022 (3)	−0.004 (4)	0.006 (3)	−0.016 (3)
O1C	0.0133 (16)	0.0168 (16)	0.0114 (15)	0.0003 (12)	−0.0011 (12)	0.0006 (12)
C1C	0.014 (3)	0.020 (3)	0.009 (3)	0.001 (3)	−0.002 (2)	0.001 (3)
C2C	0.011 (3)	0.029 (3)	0.013 (3)	0.000 (3)	0.001 (3)	−0.002 (3)
C3C	0.014 (3)	0.027 (3)	0.012 (3)	0.002 (3)	−0.002 (3)	0.002 (3)
F1C	0.017 (3)	0.042 (3)	0.032 (3)	0.009 (3)	0.005 (2)	0.004 (3)
F2C	0.016 (2)	0.048 (3)	0.014 (2)	0.001 (2)	0.0067 (19)	0.004 (2)
F3C	0.014 (2)	0.064 (4)	0.025 (3)	−0.004 (3)	−0.007 (2)	−0.008 (3)
F4C	0.037 (4)	0.046 (4)	0.014 (2)	0.002 (3)	0.000 (2)	0.010 (2)
F5C	0.024 (2)	0.048 (3)	0.017 (2)	0.007 (2)	0.0095 (19)	0.002 (2)
F6C	0.033 (3)	0.055 (3)	0.014 (2)	−0.003 (3)	−0.009 (2)	−0.002 (2)
O1D	0.0175 (17)	0.0181 (16)	0.0141 (16)	0.0006 (12)	0.0022 (12)	−0.0007 (12)
C1D	0.014 (3)	0.024 (3)	0.013 (3)	0.000 (3)	0.000 (3)	0.001 (3)
C2D	0.021 (5)	0.037 (6)	0.012 (4)	0.004 (4)	−0.003 (3)	−0.004 (4)
C3D	0.024 (4)	0.031 (4)	0.018 (3)	−0.001 (3)	0.004 (3)	0.000 (3)
F1D	0.023 (3)	0.052 (4)	0.021 (3)	0.006 (3)	0.000 (2)	0.011 (3)
F2D	0.028 (3)	0.082 (5)	0.016 (3)	0.021 (4)	0.005 (2)	−0.012 (3)
F3D	0.020 (3)	0.066 (5)	0.032 (3)	−0.010 (3)	−0.001 (3)	−0.003 (3)
F4D	0.029 (4)	0.035 (4)	0.041 (4)	−0.010 (3)	−0.006 (3)	0.003 (3)
F5D	0.034 (4)	0.076 (5)	0.026 (3)	0.001 (4)	−0.001 (3)	−0.031 (3)
F6D	0.020 (3)	0.054 (4)	0.035 (3)	0.005 (3)	−0.015 (3)	−0.006 (3)

### Geometric parameters (Å, º)

**Table:**

Fe—O1B	1.948 (6)	F1A—K1^i^	2.861 (6)
Fe—O1D	1.950 (6)	F4A—K1^i^	3.058 (7)
Fe—O1C	2.047 (6)	O1B—C1B	1.367 (11)
Fe—O1A	2.048 (6)	O1B—K1^i^	2.692 (7)
Fe—K2	3.535 (2)	C1B—C3B	1.532 (13)
Fe—K1	3.715 (2)	C1B—C2B	1.540 (13)
Fe—K1^i^	3.717 (2)	C1B—H1BA	1.0000
K1—O1D	2.674 (6)	C2B—F2B	1.323 (12)
K1—O1B^ii^	2.692 (7)	C2B—F1B	1.335 (11)
K1—F1A^ii^	2.861 (6)	C2B—F3B	1.353 (13)
K1—O1A^ii^	2.863 (7)	C3B—F6B	1.326 (13)
K1—F1C	2.877 (7)	C3B—F4B	1.331 (12)
K1—O1C	2.891 (6)	C3B—F5B	1.361 (13)
K1—F1D	3.003 (6)	F3B—K2^i^	2.888 (7)
K1—F5B^ii^	3.039 (7)	F3B—K1^i^	3.145 (7)
K1—F4A^ii^	3.058 (7)	F5B—K2^i^	2.973 (7)
K1—F4C	3.123 (6)	F5B—K1^i^	3.039 (7)
K1—F3B^ii^	3.145 (7)	O1C—C1C	1.375 (10)
K1—F4D	3.221 (7)	C1C—C2C	1.517 (12)
K2—O1A	2.689 (6)	C1C—C3C	1.522 (11)
K2—O1C	2.698 (7)	C1C—H1CA	1.0000
K2—F4D^i^	2.798 (8)	C2C—F3C	1.334 (11)
K2—F3B^ii^	2.888 (7)	C2C—F1C	1.340 (12)
K2—F5B^ii^	2.973 (7)	C2C—F2C	1.349 (10)
K2—F5C	3.032 (6)	C3C—F4C	1.324 (12)
K2—F6A	3.048 (7)	C3C—F5C	1.336 (11)
K2—F6C^iii^	3.077 (6)	C3C—F6C	1.349 (10)
K2—F1D^i^	3.088 (7)	F6C—K2^iv^	3.077 (6)
K2—K1^i^	4.395 (3)	O1D—C1D	1.356 (10)
O1A—C1A	1.381 (10)	C1D—C2D	1.528 (13)
O1A—K1^i^	2.863 (6)	C1D—C3D	1.547 (14)
C1A—C2A	1.507 (12)	C1D—H1DA	1.0000
C1A—C3A	1.524 (13)	C2D—F6D	1.325 (12)
C1A—H1AA	1.0000	C2D—F5D	1.330 (11)
C2A—F3A	1.337 (12)	C2D—F4D	1.365 (12)
C2A—F2A	1.338 (10)	C3D—F2D	1.319 (11)
C2A—F1A	1.344 (12)	C3D—F3D	1.321 (13)
C3A—F4A	1.330 (13)	C3D—F1D	1.348 (11)
C3A—F6A	1.337 (12)	F1D—K2^ii^	3.088 (7)
C3A—F5A	1.347 (11)	F4D—K2^ii^	2.798 (8)
			
O1B—Fe—O1D	143.0 (3)	F1D^i^—K2—Fe	97.66 (13)
O1B—Fe—O1C	110.7 (3)	O1A—K2—K1	80.40 (13)
O1D—Fe—O1C	94.1 (2)	O1C—K2—K1	40.37 (13)
O1B—Fe—O1A	93.6 (3)	F4D^i^—K2—K1	109.28 (15)
O1D—Fe—O1A	109.7 (3)	F3B^ii^—K2—K1	46.14 (14)
O1C—Fe—O1A	98.7 (2)	F5B^ii^—K2—K1	44.14 (14)
O1B—Fe—K2	109.49 (19)	F5C—K2—K1	72.34 (13)
O1D—Fe—K2	107.45 (19)	F6A—K2—K1	108.52 (14)
O1C—Fe—K2	49.45 (18)	F6C^iii^—K2—K1	107.35 (15)
O1A—Fe—K2	49.21 (17)	F1D^i^—K2—K1	152.62 (13)
O1B—Fe—K1	153.8 (2)	Fe—K2—K1	54.96 (4)
O1D—Fe—K1	43.83 (18)	O1A—K2—K1^i^	39.07 (14)
O1C—Fe—K1	50.70 (17)	O1C—K2—K1^i^	79.67 (13)
O1A—Fe—K1	106.50 (18)	F4D^i^—K2—K1^i^	47.01 (15)
K2—Fe—K1	73.86 (5)	F3B^ii^—K2—K1^i^	108.57 (13)
O1B—Fe—K1^i^	44.28 (19)	F5B^ii^—K2—K1^i^	153.69 (15)
O1D—Fe—K1^i^	152.21 (19)	F5C—K2—K1^i^	107.12 (14)
O1C—Fe—K1^i^	106.55 (17)	F6A—K2—K1^i^	70.74 (13)
O1A—Fe—K1^i^	49.86 (18)	F6C^iii^—K2—K1^i^	131.40 (15)
K2—Fe—K1^i^	74.57 (5)	F1D^i^—K2—K1^i^	43.05 (12)
K1—Fe—K1^i^	148.42 (4)	Fe—K2—K1^i^	54.61 (4)
O1D—K1—O1B^ii^	112.7 (2)	K1—K2—K1^i^	109.57 (4)
O1D—K1—F1A^ii^	164.9 (2)	C1A—O1A—Fe	121.7 (5)
O1B^ii^—K1—F1A^ii^	81.9 (2)	C1A—O1A—K2	123.6 (5)
O1D—K1—O1A^ii^	122.10 (19)	Fe—O1A—K2	95.6 (2)
O1B^ii^—K1—O1A^ii^	63.20 (18)	C1A—O1A—K1^i^	110.2 (5)
F1A^ii^—K1—O1A^ii^	60.00 (17)	Fe—O1A—K1^i^	97.0 (2)
O1D—K1—F1C	83.88 (19)	K2—O1A—K1^i^	104.6 (2)
O1B^ii^—K1—F1C	163.0 (2)	O1A—C1A—C2A	112.0 (7)
F1A^ii^—K1—F1C	81.81 (19)	O1A—C1A—C3A	107.5 (7)
O1A^ii^—K1—F1C	112.00 (19)	C2A—C1A—C3A	112.4 (8)
O1D—K1—O1C	63.35 (18)	O1A—C1A—H1AA	108.3
O1B^ii^—K1—O1C	123.56 (19)	C2A—C1A—H1AA	108.3
F1A^ii^—K1—O1C	112.51 (17)	C3A—C1A—H1AA	108.3
O1A^ii^—K1—O1C	170.39 (18)	F3A—C2A—F2A	106.5 (8)
F1C—K1—O1C	59.39 (18)	F3A—C2A—F1A	106.7 (8)
O1D—K1—F1D	57.24 (18)	F2A—C2A—F1A	106.3 (7)
O1B^ii^—K1—F1D	118.1 (2)	F3A—C2A—C1A	110.7 (8)
F1A^ii^—K1—F1D	113.43 (19)	F2A—C2A—C1A	113.2 (8)
O1A^ii^—K1—F1D	74.11 (18)	F1A—C2A—C1A	113.1 (8)
F1C—K1—F1D	73.39 (18)	F4A—C3A—F6A	106.6 (9)
O1C—K1—F1D	105.59 (19)	F4A—C3A—F5A	107.1 (8)
O1D—K1—F5B^ii^	115.9 (2)	F6A—C3A—F5A	106.6 (8)
O1B^ii^—K1—F5B^ii^	57.84 (19)	F4A—C3A—C1A	112.7 (8)
F1A^ii^—K1—F5B^ii^	74.45 (19)	F6A—C3A—C1A	110.3 (8)
O1A^ii^—K1—F5B^ii^	108.25 (19)	F5A—C3A—C1A	113.0 (8)
F1C—K1—F5B^ii^	112.55 (18)	C2A—F1A—K1^i^	116.1 (5)
O1C—K1—F5B^ii^	73.54 (18)	C3A—F4A—K1^i^	115.7 (6)
F1D—K1—F5B^ii^	171.23 (18)	C3A—F6A—K2	114.3 (5)
O1D—K1—F4A^ii^	112.61 (19)	C1B—O1B—Fe	132.1 (6)
O1B^ii^—K1—F4A^ii^	116.48 (19)	C1B—O1B—K1^i^	121.3 (5)
F1A^ii^—K1—F4A^ii^	54.69 (19)	Fe—O1B—K1^i^	105.4 (3)
O1A^ii^—K1—F4A^ii^	55.65 (17)	O1B—C1B—C3B	110.0 (7)
F1C—K1—F4A^ii^	56.36 (17)	O1B—C1B—C2B	108.3 (7)
O1C—K1—F4A^ii^	115.57 (18)	C3B—C1B—C2B	109.3 (8)
F1D—K1—F4A^ii^	60.14 (17)	O1B—C1B—H1BA	109.7
F5B^ii^—K1—F4A^ii^	128.35 (19)	C3B—C1B—H1BA	109.7
O1D—K1—F4C	116.14 (18)	C2B—C1B—H1BA	109.7
O1B^ii^—K1—F4C	112.7 (2)	F2B—C2B—F1B	107.9 (9)
F1A^ii^—K1—F4C	57.82 (16)	F2B—C2B—F3B	106.6 (9)
O1A^ii^—K1—F4C	117.47 (17)	F1B—C2B—F3B	105.5 (8)
F1C—K1—F4C	53.42 (18)	F2B—C2B—C1B	113.9 (8)
O1C—K1—F4C	54.68 (16)	F1B—C2B—C1B	110.3 (8)
F1D—K1—F4C	126.43 (19)	F3B—C2B—C1B	112.1 (8)
F5B^ii^—K1—F4C	60.50 (18)	F6B—C3B—F4B	107.3 (8)
F4A^ii^—K1—F4C	83.19 (19)	F6B—C3B—F5B	106.3 (9)
O1D—K1—F3B^ii^	71.22 (18)	F4B—C3B—F5B	106.5 (9)
O1B^ii^—K1—F3B^ii^	54.89 (18)	F6B—C3B—C1B	110.3 (9)
F1A^ii^—K1—F3B^ii^	122.69 (19)	F4B—C3B—C1B	114.2 (9)
O1A^ii^—K1—F3B^ii^	115.15 (17)	F5B—C3B—C1B	111.8 (8)
F1C—K1—F3B^ii^	132.85 (19)	C2B—F3B—K2^i^	122.8 (6)
O1C—K1—F3B^ii^	73.62 (18)	C2B—F3B—K1^i^	114.0 (6)
F1D—K1—F3B^ii^	119.30 (17)	K2^i^—F3B—K1^i^	92.40 (19)
F5B^ii^—K1—F3B^ii^	51.94 (17)	C3B—F5B—K2^i^	126.5 (6)
F4A^ii^—K1—F3B^ii^	170.79 (18)	C3B—F5B—K1^i^	112.8 (6)
F4C—K1—F3B^ii^	102.85 (19)	K2^i^—F5B—K1^i^	92.91 (19)
O1D—K1—F4D	55.11 (18)	C1C—O1C—Fe	122.8 (5)
O1B^ii^—K1—F4D	72.43 (19)	C1C—O1C—K2	124.2 (5)
F1A^ii^—K1—F4D	130.92 (18)	Fe—O1C—K2	95.3 (2)
O1A^ii^—K1—F4D	71.10 (18)	C1C—O1C—K1	111.1 (5)
F1C—K1—F4D	122.75 (18)	Fe—O1C—K1	96.1 (2)
O1C—K1—F4D	116.56 (17)	K2—O1C—K1	102.4 (2)
F1D—K1—F4D	51.80 (18)	O1C—C1C—C2C	111.8 (7)
F5B^ii^—K1—F4D	120.40 (19)	O1C—C1C—C3C	107.6 (7)
F4A^ii^—K1—F4D	101.14 (19)	C2C—C1C—C3C	111.9 (7)
F4C—K1—F4D	171.16 (18)	O1C—C1C—H1CA	108.5
F3B^ii^—K1—F4D	73.87 (18)	C2C—C1C—H1CA	108.5
O1A—K2—O1C	70.43 (17)	C3C—C1C—H1CA	108.5
O1A—K2—F4D^i^	80.6 (2)	F3C—C2C—F1C	107.1 (8)
O1C—K2—F4D^i^	69.08 (19)	F3C—C2C—F2C	106.3 (7)
O1A—K2—F3B^ii^	69.64 (18)	F1C—C2C—F2C	105.8 (7)
O1C—K2—F3B^ii^	80.8 (2)	F3C—C2C—C1C	114.5 (7)
F4D^i^—K2—F3B^ii^	143.3 (2)	F1C—C2C—C1C	112.9 (8)
O1A—K2—F5B^ii^	119.22 (19)	F2C—C2C—C1C	109.8 (7)
O1C—K2—F5B^ii^	77.4 (2)	F4C—C3C—F5C	107.0 (8)
F4D^i^—K2—F5B^ii^	132.1 (2)	F4C—C3C—F6C	106.9 (8)
F3B^ii^—K2—F5B^ii^	55.06 (17)	F5C—C3C—F6C	106.5 (7)
O1A—K2—F5C	122.54 (18)	F4C—C3C—C1C	113.0 (7)
O1C—K2—F5C	55.93 (17)	F5C—C3C—C1C	109.9 (7)
F4D^i^—K2—F5C	63.32 (19)	F6C—C3C—C1C	113.2 (8)
F3B^ii^—K2—F5C	115.89 (19)	C2C—F1C—K1	116.7 (5)
F5B^ii^—K2—F5C	69.82 (18)	C3C—F4C—K1	115.3 (5)
O1A—K2—F6A	56.03 (17)	C3C—F5C—K2	114.6 (5)
O1C—K2—F6A	123.39 (17)	C3C—F6C—K2^iv^	149.4 (6)
F4D^i^—K2—F6A	114.6 (2)	C1D—O1D—Fe	130.7 (6)
F3B^ii^—K2—F6A	65.49 (19)	C1D—O1D—K1	121.9 (5)
F5B^ii^—K2—F6A	112.2 (2)	Fe—O1D—K1	105.8 (2)
F5C—K2—F6A	177.8 (2)	O1D—C1D—C2D	109.6 (8)
O1A—K2—F6C^iii^	170.4 (2)	O1D—C1D—C3D	109.1 (7)
O1C—K2—F6C^iii^	111.62 (18)	C2D—C1D—C3D	110.9 (8)
F4D^i^—K2—F6C^iii^	91.4 (2)	O1D—C1D—H1DA	109.1
F3B^ii^—K2—F6C^iii^	119.7 (2)	C2D—C1D—H1DA	109.1
F5B^ii^—K2—F6C^iii^	70.11 (19)	C3D—C1D—H1DA	109.1
F5C—K2—F6C^iii^	56.78 (16)	F6D—C2D—F5D	108.1 (8)
F6A—K2—F6C^iii^	124.30 (18)	F6D—C2D—F4D	106.2 (8)
O1A—K2—F1D^i^	75.14 (17)	F5D—C2D—F4D	106.5 (9)
O1C—K2—F1D^i^	117.56 (18)	F6D—C2D—C1D	111.1 (9)
F4D^i^—K2—F1D^i^	54.91 (17)	F5D—C2D—C1D	113.4 (9)
F3B^ii^—K2—F1D^i^	131.32 (18)	F4D—C2D—C1D	111.1 (7)
F5B^ii^—K2—F1D^i^	163.23 (19)	F2D—C3D—F3D	107.8 (9)
F5C—K2—F1D^i^	111.10 (18)	F2D—C3D—F1D	106.4 (8)
F6A—K2—F1D^i^	67.20 (18)	F3D—C3D—F1D	106.5 (8)
F6C^iii^—K2—F1D^i^	96.01 (18)	F2D—C3D—C1D	114.3 (8)
O1A—K2—Fe	35.22 (13)	F3D—C3D—C1D	110.1 (8)
O1C—K2—Fe	35.22 (12)	F1D—C3D—C1D	111.4 (8)
F4D^i^—K2—Fe	71.94 (15)	C3D—F1D—K1	115.1 (6)
F3B^ii^—K2—Fe	71.37 (14)	C3D—F1D—K2^ii^	121.0 (6)
F5B^ii^—K2—Fe	99.10 (15)	K1—F1D—K2^ii^	92.36 (18)
F5C—K2—Fe	89.38 (11)	C2D—F4D—K2^ii^	130.0 (6)
F6A—K2—Fe	89.56 (12)	C2D—F4D—K1	110.6 (5)
F6C^iii^—K2—Fe	146.14 (14)	K2^ii^—F4D—K1	93.5 (2)
			
Fe—O1A—C1A—C2A	58.2 (9)	K1—O1C—C1C—C2C	−52.8 (8)
K2—O1A—C1A—C2A	−178.7 (5)	Fe—O1C—C1C—C3C	−177.0 (5)
K1^i^—O1A—C1A—C2A	−54.1 (8)	K2—O1C—C1C—C3C	−52.3 (8)
Fe—O1A—C1A—C3A	−177.8 (6)	K1—O1C—C1C—C3C	70.5 (7)
K2—O1A—C1A—C3A	−54.7 (8)	O1C—C1C—C2C—F3C	−175.2 (7)
K1^i^—O1A—C1A—C3A	69.8 (7)	C3C—C1C—C2C—F3C	64.0 (10)
O1A—C1A—C2A—F3A	−57.5 (10)	O1C—C1C—C2C—F1C	61.9 (10)
C3A—C1A—C2A—F3A	−178.7 (8)	C3C—C1C—C2C—F1C	−58.9 (10)
O1A—C1A—C2A—F2A	−177.0 (7)	O1C—C1C—C2C—F2C	−55.9 (10)
C3A—C1A—C2A—F2A	61.8 (10)	C3C—C1C—C2C—F2C	−176.7 (7)
O1A—C1A—C2A—F1A	62.1 (10)	O1C—C1C—C3C—F4C	−60.8 (10)
C3A—C1A—C2A—F1A	−59.1 (11)	C2C—C1C—C3C—F4C	62.4 (10)
O1A—C1A—C3A—F4A	−60.4 (10)	O1C—C1C—C3C—F5C	58.6 (9)
C2A—C1A—C3A—F4A	63.3 (10)	C2C—C1C—C3C—F5C	−178.2 (8)
O1A—C1A—C3A—F6A	58.6 (10)	O1C—C1C—C3C—F6C	177.5 (8)
C2A—C1A—C3A—F6A	−177.7 (8)	C2C—C1C—C3C—F6C	−59.3 (10)
O1A—C1A—C3A—F5A	177.9 (8)	F3C—C2C—F1C—K1	−162.6 (5)
C2A—C1A—C3A—F5A	−58.4 (11)	F2C—C2C—F1C—K1	84.4 (7)
F3A—C2A—F1A—K1^i^	87.7 (7)	C1C—C2C—F1C—K1	−35.7 (8)
F2A—C2A—F1A—K1^i^	−159.0 (5)	F5C—C3C—F4C—K1	−100.1 (6)
C1A—C2A—F1A—K1^i^	−34.2 (10)	F6C—C3C—F4C—K1	146.1 (5)
F6A—C3A—F4A—K1^i^	−100.7 (7)	C1C—C3C—F4C—K1	20.9 (9)
F5A—C3A—F4A—K1^i^	145.5 (6)	F4C—C3C—F5C—K2	82.7 (7)
C1A—C3A—F4A—K1^i^	20.5 (9)	F6C—C3C—F5C—K2	−163.3 (5)
F4A—C3A—F6A—K2	84.6 (8)	C1C—C3C—F5C—K2	−40.3 (9)
F5A—C3A—F6A—K2	−161.2 (6)	F4C—C3C—F6C—K2^iv^	50.4 (15)
C1A—C3A—F6A—K2	−38.1 (9)	F5C—C3C—F6C—K2^iv^	−63.7 (14)
Fe—O1B—C1B—C3B	−111.6 (8)	C1C—C3C—F6C—K2^iv^	175.4 (8)
K1^i^—O1B—C1B—C3B	54.1 (9)	Fe—O1D—C1D—C2D	130.2 (7)
Fe—O1B—C1B—C2B	129.0 (7)	K1—O1D—C1D—C2D	−66.0 (9)
K1^i^—O1B—C1B—C2B	−65.4 (8)	Fe—O1D—C1D—C3D	−108.2 (8)
O1B—C1B—C2B—F2B	173.4 (8)	K1—O1D—C1D—C3D	55.6 (9)
C3B—C1B—C2B—F2B	53.6 (11)	O1D—C1D—C2D—F6D	−59.5 (10)
O1B—C1B—C2B—F1B	−65.1 (10)	C3D—C1D—C2D—F6D	179.9 (7)
C3B—C1B—C2B—F1B	175.1 (8)	O1D—C1D—C2D—F5D	178.5 (8)
O1B—C1B—C2B—F3B	52.2 (10)	C3D—C1D—C2D—F5D	58.0 (11)
C3B—C1B—C2B—F3B	−67.6 (10)	O1D—C1D—C2D—F4D	58.5 (10)
O1B—C1B—C3B—F6B	60.7 (10)	C3D—C1D—C2D—F4D	−62.0 (10)
C2B—C1B—C3B—F6B	179.4 (9)	O1D—C1D—C3D—F2D	−174.5 (9)
O1B—C1B—C3B—F4B	−178.5 (8)	C2D—C1D—C3D—F2D	−53.7 (11)
C2B—C1B—C3B—F4B	−59.7 (11)	O1D—C1D—C3D—F3D	64.0 (10)
O1B—C1B—C3B—F5B	−57.4 (11)	C2D—C1D—C3D—F3D	−175.2 (7)
C2B—C1B—C3B—F5B	61.4 (11)	O1D—C1D—C3D—F1D	−53.9 (10)
F2B—C2B—F3B—K2^i^	−36.4 (10)	C2D—C1D—C3D—F1D	66.9 (10)
F1B—C2B—F3B—K2^i^	−151.0 (5)	F2D—C3D—F1D—K1	154.9 (6)
C1B—C2B—F3B—K2^i^	88.9 (8)	F3D—C3D—F1D—K1	−90.3 (8)
F2B—C2B—F3B—K1^i^	−146.4 (6)	C1D—C3D—F1D—K1	29.8 (9)
F1B—C2B—F3B—K1^i^	99.1 (7)	F2D—C3D—F1D—K2^ii^	45.4 (10)
C1B—C2B—F3B—K1^i^	−21.1 (9)	F3D—C3D—F1D—K2^ii^	160.2 (5)
F6B—C3B—F5B—K2^i^	161.5 (6)	C1D—C3D—F1D—K2^ii^	−79.8 (8)
F4B—C3B—F5B—K2^i^	47.3 (10)	F6D—C2D—F4D—K2^ii^	−153.1 (5)
C1B—C3B—F5B—K2^i^	−78.1 (9)	F5D—C2D—F4D—K2^ii^	−38.1 (11)
F6B—C3B—F5B—K1^i^	−86.2 (8)	C1D—C2D—F4D—K2^ii^	85.9 (9)
F4B—C3B—F5B—K1^i^	159.6 (6)	F6D—C2D—F4D—K1	93.2 (7)
C1B—C3B—F5B—K1^i^	34.2 (9)	F5D—C2D—F4D—K1	−151.8 (6)
Fe—O1C—C1C—C2C	59.7 (9)	C1D—C2D—F4D—K1	−27.8 (9)
K2—O1C—C1C—C2C	−175.5 (5)		

Symmetry codes: (i) −*x*+1, *y*−1/2, −*z*+1/2; (ii) −*x*+1, *y*+1/2, −*z*+1/2; (iii) *x*+1/2, −*y*+1/2, −*z*; (iv) *x*−1/2, −*y*+1/2, −*z*.

### Hydrogen-bond geometry (Å, º)

**Table:**

*D*—H···*A*	*D*—H	H···*A*	*D*···*A*	*D*—H···*A*
C1*A*—H1*AA*···F3*B*^ii^	1.00	2.54	3.236 (10)	126
C1*B*—H1*BA*···F2*C*	1.00	2.56	3.199 (11)	122
C1*C*—H1*CA*···F4*D*^i^	1.00	2.56	3.201 (11)	122
C1*D*—H1*DA*···F2*A*^v^	1.00	2.46	3.361 (11)	150
C1*D*—H1*DA*···F3*A*	1.00	2.42	3.093 (11)	124

Symmetry codes: (i) −*x*+1, *y*−1/2, −*z*+1/2; (ii) −*x*+1, *y*+1/2, −*z*+1/2; (v) *x*−1/2, −*y*+1/2, −*z*+1.
